# Lifetime prediction for organic coating under alternating hydrostatic pressure by artificial neural network

**DOI:** 10.1038/srep40827

**Published:** 2017-01-17

**Authors:** Wenliang Tian, Fandi Meng, Li Liu, Ying Li, Fuhui Wang

**Affiliations:** 1 Institute of Metal research, Chinese Academy of Science, Wencui Rd 62, Shenyang, 110016, China

## Abstract

A concept for prediction of organic coatings, based on the alternating hydrostatic pressure (AHP) accelerated tests, has been presented. An AHP accelerated test with different pressure values has been employed to evaluate coating degradation. And a back-propagation artificial neural network (BP-ANN) has been established to predict the service property and the service lifetime of coatings. The pressure value (*P*), immersion time (*t*) and service property (impedance modulus |*Z*|) are utilized as the parameters of the network. The average accuracies of the predicted service property and immersion time by the established network are 98.6% and 84.8%, respectively. The combination of accelerated test and prediction method by BP-ANN is promising to evaluate and predict coating property used in deep sea.

Organic coatings used in deep sea environment are confronted with much tough challenges and serious deteriorations. One of the most important considerations for the coating technology is to evaluate coating performance and predict coating failure at the earliest time in the life cycle of the coating. The laboratory accelerated tests^1^,^2^,^3^,^4^,^5^,^6^ have been adopted widely to avoid long-lasting experiments, especially for the newly developed materials or the novel serving conditions. By simulating the field conditions, the lifetime of the test specimens is reduced significantly if the stresses are applied at an increased level. Then, the relationship between the increased stress level and the lifetime of the test specimens is analyzed to deduce the lifetime at field conditions.

The lifetime prediction can be made based on the laboratory accelerated tests, which can prevent unexpected failures and minimize overall maintenance costs effectively. Various methods or algorithms for the lifetime prediction of materials have been developed. Existing life prediction methods can be roughly classified into two aspects: (1) the model-based (or physics-based) methods, and (2) the data-driven methods. The model-based methods^7^,^8^,^9^,^10^,^11^ predict the remaining useful life mainly by using the damage propagation models which are based on the damage mechanics. If properly used, the model-based methods can make quite accurate predictions. However, the condition stresses on materials and the damage propagation processes of materials are typically very complex, and the mechanical and physical insight into the failure behavior of materials is relative limited at the initial stage of investigations, so the authentic physics-based models are very difficult to build. The data-driven method^12^,^13^,^14^,^15^ utilizes the collected condition monitoring data for lifetime prediction without the establishment of complex physical models. It is based on the understanding that the condition monitoring data are associated with the extracted features during the degradation process. The data-driven methods aim at modeling the relationship of the condition monitoring data, the material property after degradation and the lifetime. By the analysis of the dependencies between the monitoring data and the material property, the service property or lifetime of materials can be predicted. As the difficulty for the establishment of physics-based models, the data-driven methods have caused increasing attention for the evaluation and prediction of materials, especially for the new materials and the novel serving conditions.

Artificial neural network (ANN), as an effective data-driven modeling method, has been considered to be a very promising tool for evaluation and prediction of materials because of its self-learning ability, nonlinearity and arbitrary function approximation ability^16^,^17^,^18^. The ANN is a simplification and simulation of the bio-neural network. As a model that based on empirical data, it can readily deal with some odd, incomplete or inconsistent data. Tian *et al*.^19^ developed an ANN approach which utilized age and condition monitoring data as inputs and the life percentage as output, and the trained ANN, which used both the suspension history and the failure history, could be used for remaining useful life prediction. Cottis *et al*.^20^ presented neural network methods to model the pitting corrosion behavior of a stainless steel as a function of solution composition and temperature. Lee *et al*.^21^ proposed two artificial networks for classification of polymer coating quality, and excellent agreement between the predictions made by experienced operators and the classification results obtained from the ANNs was obtained. Xiang *et al*.^22^ established a BP-ANN model to predict fatigue property of natural rubber composites, in which three mechanical properties and viscoelasticity property of the composites were utilized as the input vectors while fatigue property (tensile fatigue life) as the output vector. And a quite accurate prediction of the ANN was obtained. The ANN can readily address material problems that are analytical difficult and for which conventional approaches are not practical. And considering the complexity of coating failure, the non-linear analysis method, ANN, is suitable for the evaluation and prediction of organic coatings as a data-driven modelling method.

Based on our previous works^23^,^24^,^25^, the alternating hydrostatic pressure (AHP) decreases the protective properties and accelerates the failure of coatings. The coatings used in deep sea are confronted with much more severe conditions. Meanwhile, the influence of different alternating pressure values are still unknown, as well as the lifetime prediction for coatings applied in deep sea. This study first presents and compares the influence of different alternating pressure values on coating degradation as characterized by electrochemical impedance spectroscopy (EIS) measurement as the main approach. And then a back-propagation artificial neural network (BP-ANN) model, which consists of immersion time (*t*), pressure value (*P*) of the test conditions and impedance modulus (|*Z*|) of the coating, has been established by Matlab software. The data are obtained from the accelerated alternating pressure tests when exposed to the AHP at *P* = 1 atm (atmospheric pressure), 35 atm, 60 atm, 81 atm and 100 atm, respectively. The impedance modulus of the coating are the impedance values at the lowest frequency (*f* = 0.01 Hz). The total impedance modulus reflects the systematic properties of the coating/steel system, since it not only includes the coating resistance but also the charge transfer resistance in the interface as well as all other resistances in the system, and thus it can be selected as the indicator for coating performance^26^,^27^,^28^. By training the network with specified inputs and outputs, we hope to predict the service property (the impedance modulus of the coating) or the service lifetime of the coating. And then the trained network can make predictions for coatings under any alternating pressure.

## Results and Discussion

### AHP accelerated test results

**Figure 1:**
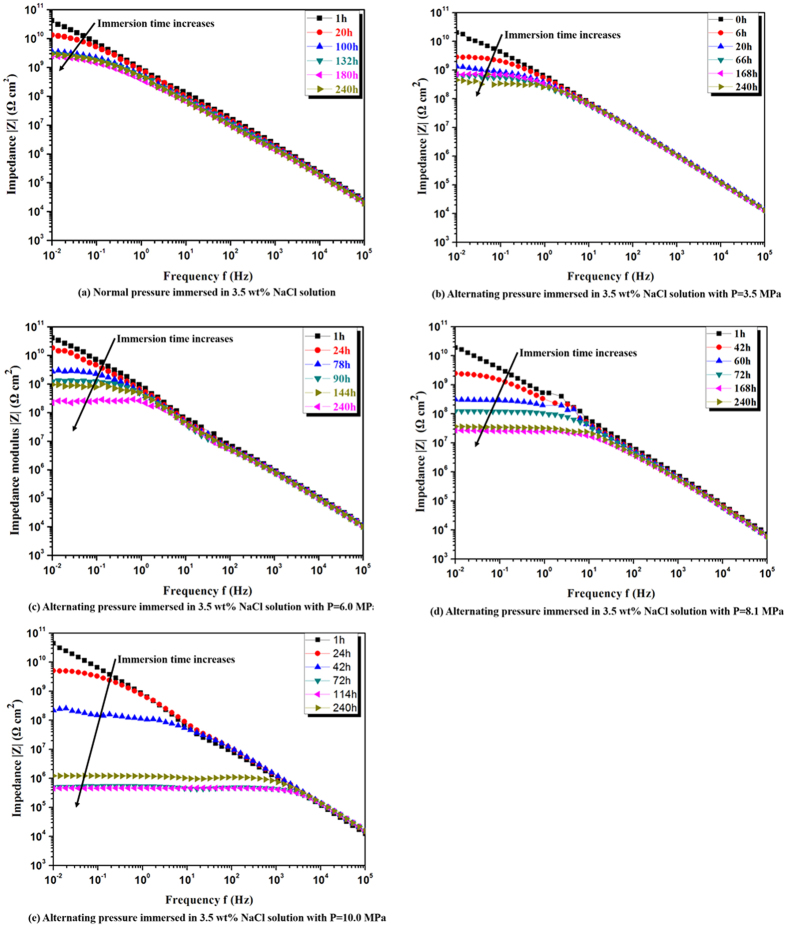
Impedance modulus as a function of frequency for coating/steel system immersed in 3.5 wt.% NaCl solution with pressure values: (**a**) *P* = 1 atm (atmospheric immersion); (**b**) *P* = 35 atm; (**c**) *P* = 60 atm; (**d**) *P* = 81 atm and (**e**) *P* = 100 atm of AHP.

To characterize the barrier properties of organic coatings, EIS data were collected for coating/steel samples immersed in 3.5 wt.% NaCl solution under different pressure values of AHP. As shown in Fig. 1a for atmospheric immersion, the impedance modulus |*Z*| was plotted as a function of the frequency in EIS tests as well as the immersion time. Similar figures for samples immersed under AHP at pressure values of *P* = 35 atm, 60 atm, 81 atm and 100 atm were presented in Fig. 1b–e.

For all pressure values, the low frequency impedance modulus decreases with immersion time, while at higher frequencies the impedance modulus appears independent on the immersion time. This behavior demonstrates that barrier property of organic coatings is deteriorated by short-term (10 days in our work) immersion under AHP. Due to the good quality of the coating samples which have no pinholes or air bubbles, the decrease of impedance modulus mainly indicates water uptake into the coating layer^29^,^30^.

**Figure 2:**
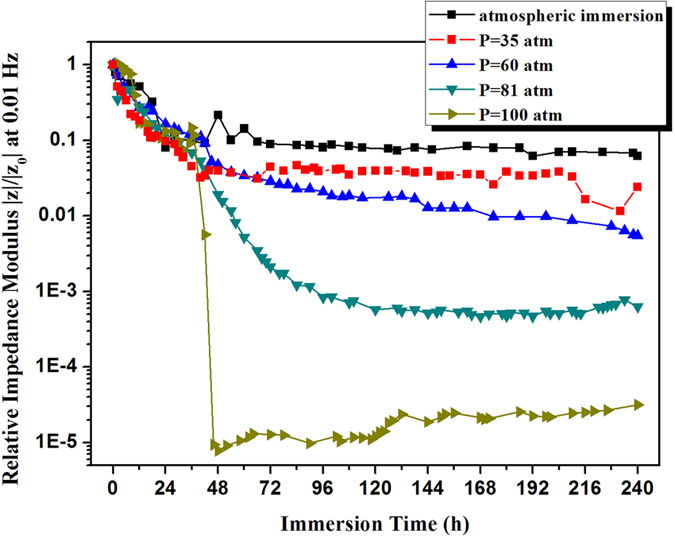
Relative impedance modulus at low frequency (*f* = 0.01 Hz) |Z| as a function of immersion time for coating/steel samples immersed in 3.5 wt.% NaCl solution with pressure values *P* = 1 atm (atmospheric immersion), 35 atm, 60 atm, 81 atm and 100 atm of AHP.

The impedance modulus (|*Z*|) at low frequency (*f* = 0.01 Hz) serves as a strong indicator of the corrosion resistance of coating samples^26^,^31^,^32^. The impedance values at initial and final immersion under different pressure values have been listed in Table 1. For coatings exposed to AP immersion, |*Z*| declines from 4.07 × 10^10^ to 1.9 × 10^9^ Ω·cm^2^ after being immersed for 240 h. While exposed to AHP environment, |*Z*| declines more rapidly, from 2.04 × 10^10^ to 4.95 × 10^8^ Ω·cm^2^, 7.24 × 10^10^ to 9.75 × 10^7^ Ω·cm^2^, from 5.89 × 10^10^ to 3.68 × 10^7^ Ω·cm^2^ and from 3.89 × 10^10^ to 1.23 × 10^6^ Ω·cm^2^, respectively for *P* = 35 atm, 60 atm, 81 atm and 100 atm. To illustrate the change in the coating’s barrier property over time, a plot of the relative low-frequency (*f* = 0.01 Hz) impedance modulus under different pressure values of AHP as a function of immersion time is presented in Fig. 2. To avoid the influence of coating thickness on coating’s barrier property, the relative low-frequency impedance modulus is adopted. The relative low-frequency impedance modulus is obtained by normalizing the low-frequency impedance modulus with the modulus at initial immersion. The decrease rate of relative low-frequency impedance modulus is substantial at the early stage of immersion followed by a relatively slower decrease. The decrease is more pronounced for AHP environment than atmospheric immersion. Moreover, the higher pressure value of AHP further accelerates the decrease of the low-frequency impedance modulus. The high decrease rate reveals the AHP enhances the water permeation into the coating, thus the degradation process of organic coatings is accelerated by the AHP.

### Prediction of the service property based on the trained network

To verify the ability of the artificial neural network (ANN) in coating lifetime prediction, we apply experimental data of the previous accelerated test to the network. Immersion time (*t*), pressure value (*P*) of the test conditions and impedance modulus (|*Z*|) of the coating have been selected as the network parameters, as listed in Table 2. At first we apply the immersion time and pressure value as input parameters and the impedance modulus (the service property) as the output parameter of the network, and the service property could be predicted according to the trained network.

**Figure 3:**
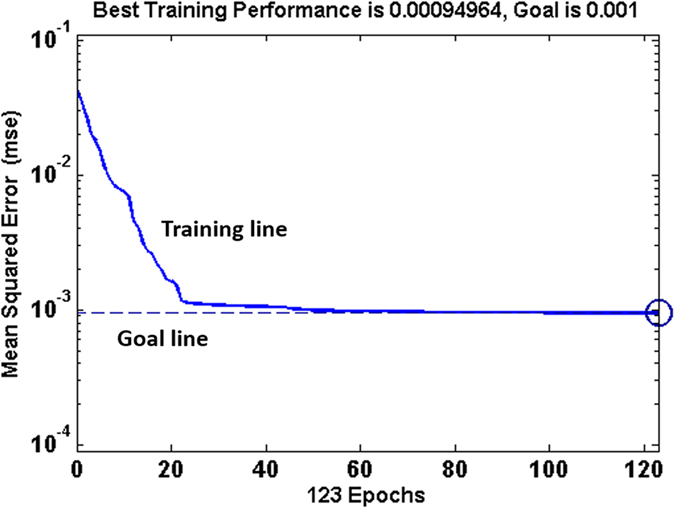
Training curve of the BP neural network model for serving property prediction of organic coatings.

The training process of BP-ANN for property prediction is shown in Fig. 3. After 123 iterations of training, the error is 0.00094964, less than the error goal 0.001, demonstrating that the established network can reach the error goal.

**Figure 4:**
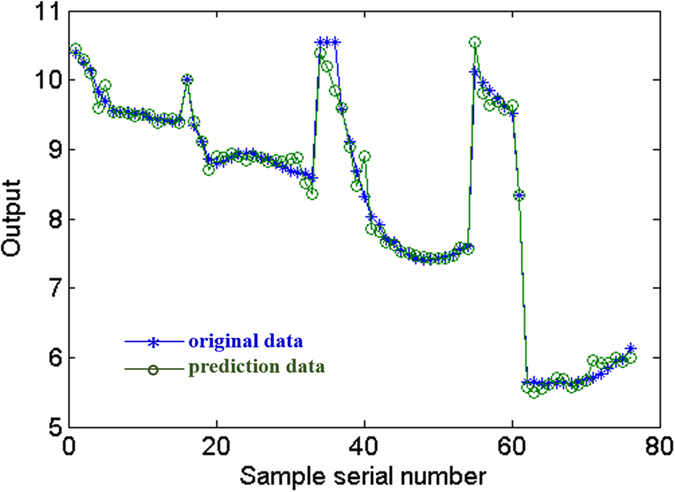
Training results of the BP-ANN for serving property prediction of coatings.

In order to validate the training effect of the above network, we compare the predicted data and experimental data of the training samples, and the results are shown in Fig. 4. It shows a high ability of the network to produce outputs for the inputs used in the training process. The figure shows a high correlation between the predicted and target outputs, and the errors between the predicted and experimental data are small. The average training accuracy is 97.7%, indicating that the established network could be used for validation data in the following analysis.

**Figure 5:**
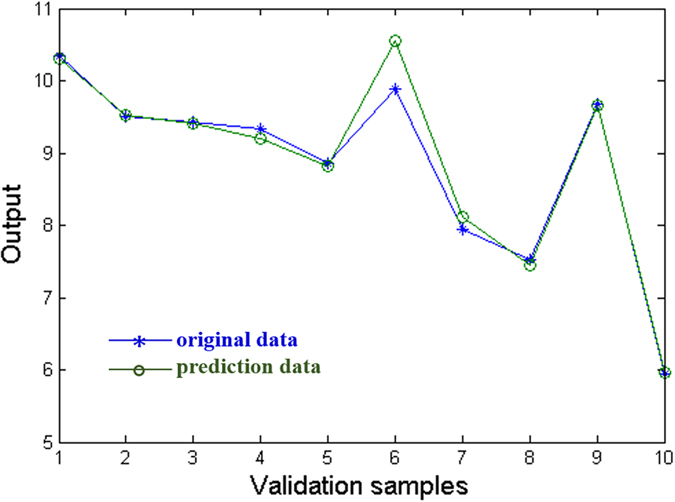
Prediction results of the BP-ANN for serving property prediction of coatings.

For generalization ability, we investigate its ability to respond to the ten validation data which are not included in the training process. The results predicted by the above established network are shown in Fig. 5. The prediction results of the data are listed in Table 3. Obviously, the errors between the predicted data and the targets are quite small and a high correlation between the predicted data and targets is clearly visible. The average accuracy of the validation data is 98.6%, indicating that the established network is effective and feasible.

According to the high accuracy of the predicted data both in the training process and validation process, it can be concluded that the proposed neural network is capable of predicting of the service property of the organic coating.

### Prediction of the lifetime based on the trained network

Then we apply the pressure value and impedance modulus as input parameters and the immersion time as the output parameter of the network, and the coating lifetime could be predicted according to the trained network.

**Figure 6:**
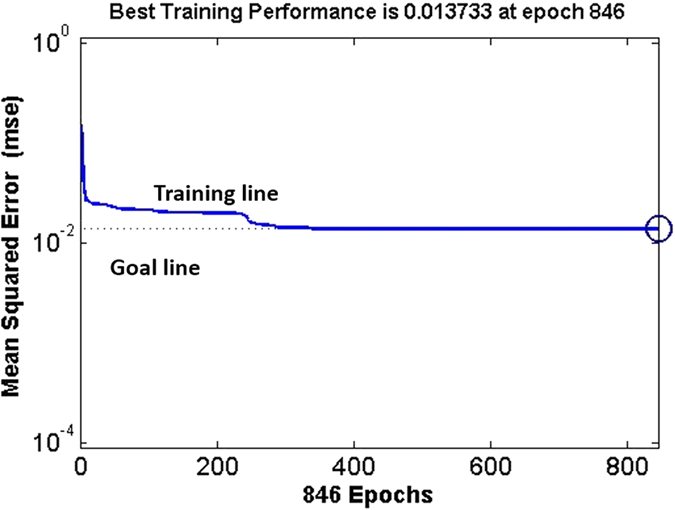
Training curve of the BP neural network model for life prediction of coatings.

The training process of BP-ANN for lifetime prediction is shown in Fig. 6. After 846 iterations of training, the training error is 0.0137.

**Figure 7:**
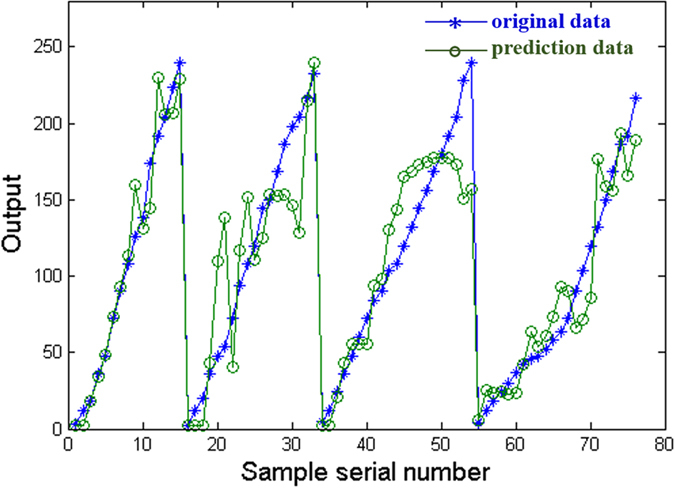
Training results of the BP-ANN for life prediction of coatings.

Figure 7 exhibits the predicted data and experimental data of the training samples. The figure shows a high correlation between the predicted data and targets, and the errors between the predicted and actual data are small. And the average training accuracy is 90.4%, indicating that the established network could be used for validation data in the following analysis.

**Figure 8:**
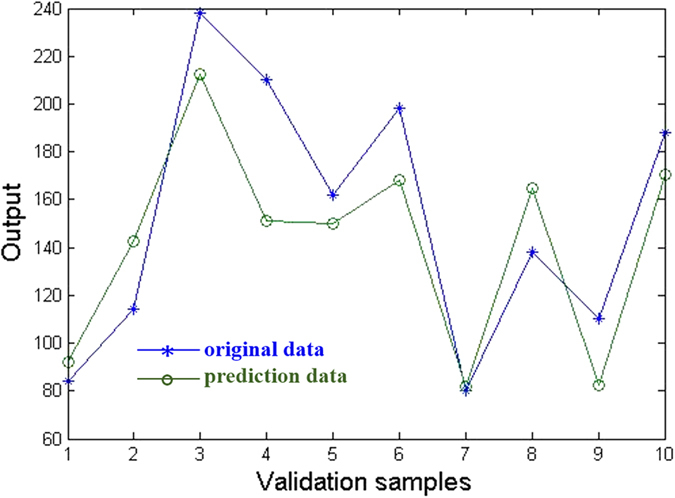
Prediction results of the BP-ANN for life prediction of coatings.

The other ten validation data, which are not included in the training process, are utilized to check the generalization ability of the network. The results predicted by the above established network are shown in Fig. 8. The prediction results of the validation data are listed in Table 4. The errors between the predicted data and the experimental data are small. The average accuracy of the validation samples is 84.8%.

To the best of our knowledge, this study is the first to give a clear picture about the prediction of organic coatings used in deep sea. The combination of laboratory accelerated tests and prediction method by artificial neural network is promising to evaluate service properties of coatings in the novel serving environments. And this technique can not only be applied to make predictions for organic coatings under deep sea, but also provides a new potential way for corrosion research into other materials and other serving environments.

### Prediction for another condition of AHP tests based on the trained network

**Figure 9:**
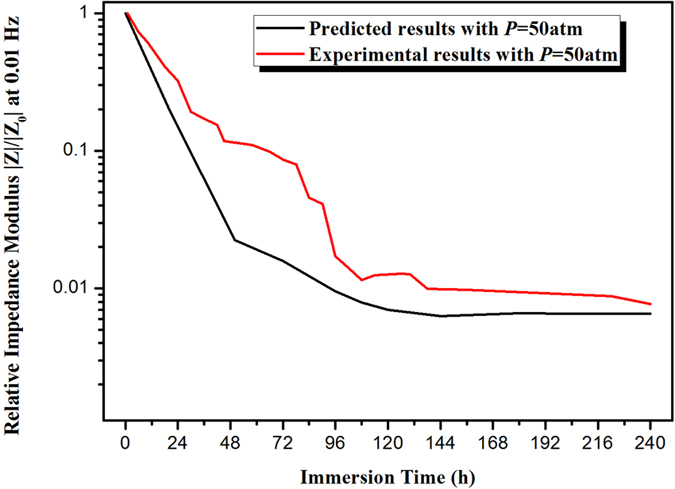
The experimental data and predicted data (relative impedance modulus) as a function of immersion time with pressure value *P* = 50 atm.

To verify whether the trained network can make predictions under any alternating pressure of AHP tests, the coating exposed to *P* = 50 atm of AHP was studied by EIS. And the experimental data and predicted data are shown in Fig. 9. The black line represents the predicted results based on the trained network and the red line is the experimental results. The figure shows a high correlation between the experimental data and predicted data. And the average training accuracy is 93.3%, indicating that the established network could be used to make predictions for coatings under any alternating pressure of AHP tests.

## Summary

A back-propagation artificial neural network model, which consists of pressure value, lifetime (immersion time) and service property (impedance modulus) of coatings, has been established to predict the service property or the service lifetime of the organic coating under AHP, and the average accuracies for the predicted service property and service time are 98.6% and 84.8%, respectively. Results indicate that the combination of laboratory accelerated tests and prediction method by neural network is promising to make evaluation and prediction of degradation for organic coatings under AHP.

## Methods

### Sample preparation

The high solid epoxy coating (HSEC) used in this work was a commercial solvent based paint, which is a two-component (A and B), epoxy-polyamide, heavy-duty protective organic coatings (H44-61, Xiamen Sunrui Ship Coating Co., Ltd, China) used in marine environments. Component A is binder and Component B is curing agent. HX-501 was used as lacquer diluent, and it is a mixture of xylene n-butanol at the ratio of 10:3. Then Component A was mixed with Component B at the ratio of 7.1:1. After thorough mixing of the two components, the coating was applied on the steel by hand brushing. In order to obtain a uniform coating, the samples were cured in an oven under the following conditions: 40 °C for 24 h and room temperature (25 °C, 30% RH) for 168 h (7 days). The average coating thickness was measured by a handheld electronic gauge (PosiTector 6000 of Defelsko, United States) according to ISO 2808-2007^33^. The thickness of the coating was controlled to 250 ± 10 μm. Prior to all the tests, the samples were stored in a desiccator to keep dry and to avoid any changes in properties due to adsorption of moisture from the atmosphere.

### The device for AHP accelerated test

**Figure 10:**
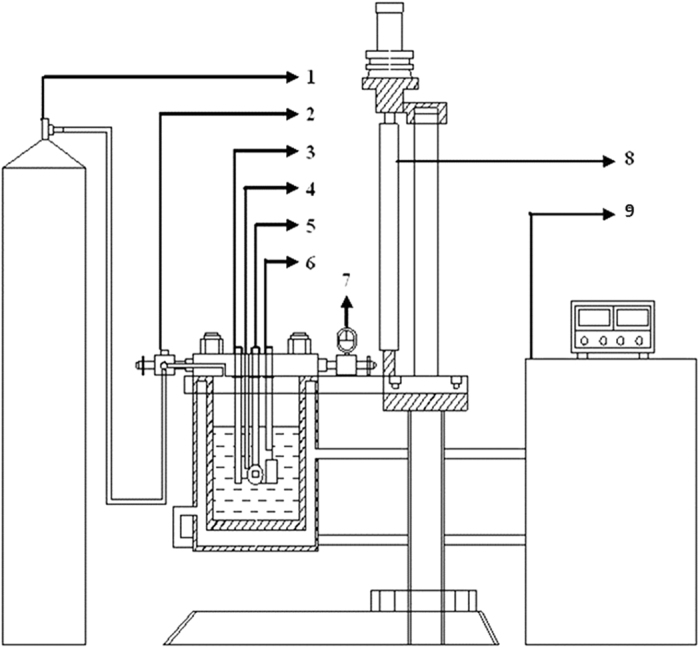
Schematic diagram of deep ocean simulation device: (1) nitrogen cylinder; (2) valve; (3) solid reference electrode; (4) thermocouple; (5) working electrode; (6) counter electrode; (7) pressure meter; (8) automatic elevator and (9) temperature controller.

Experiments were carried out in an Automatic Deep Ocean Simulation System (shown in Fig. 10) and details of the setup have been reported in ref. 25. The system includes autoclave, hydrostatic system, pneumatic system, thermostatic system and monitoring system. The high hydrostatic pressure was obtained by pushing nitrogen into the autoclave of the system, which was controlled by a pressure valve. The high hydrostatic pressure in the autoclave was kept at 35 atm, 60 atm, 81 atm and 100 atm, respectively (which are equivalent to 350 m, 600 m, 810 m and 1000 m depth hydrostatic pressure in the ocean), and the low pressure was kept as 1 atm (which is the atmospheric pressure). One alternating pressure cycle contained high pressure for 6 h and low pressure for 6 h. Every experimental process lasted for 240 h (10 days), consisting of 20 alternating cycles. The 3.5 wt.% NaCl solution was applied as the test solution. The solution temperature was controlled and monitored at room temperature (25 ± 1 °C) by the thermostatic system. With this experimental setup, *in situ* electrochemical measurements under different high pressure values of AHP could be conducted.

### EIS measurements

An AUTOLAB electrochemical station (Metrohm, Switzerland) was used for EIS measurements in this work. A conventional three-electrode system was used, i.e. with the coating/steel system as the working electrode, platinum plate (20 mm × 20 mm) as the counter electrode and the solid Ag/AgCl electrode (saturated with KCl, *E*_(vs. SCE)_ = −0.157 V) as the reference electrode. The electrodes were mounted in the specific positions of the autoclave and sealed to keep contact. The EIS tests were performed over a frequency ranging from 100 KHz to 10 mHz. According to the highly resistive system (as high as 10^11^ Ω • cm^2^) before immersion and the rapid decrease in resistance due to alternating pressure^25^, a sinusoidal AC perturbation of 50 mV (rms) amplitude coupled with the open circuit potential (OCP) was applied to the coating/steel system. Considering the possible perturbation effect of alternating pressure, the EIS test was conducted after the OCP got a steady value (less than 10 min after pressurization or depressurization process) and was effectuated every hour during experiments. And the EIS test was repeated under different high pressure values of AHP.

### Back-propagation artificial neural network (BP-ANN)

**Figure 11:**
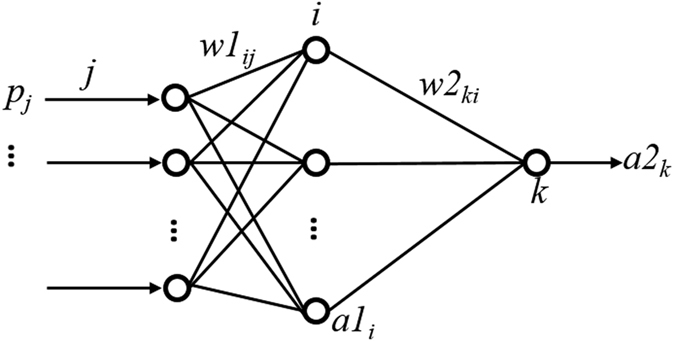
Structure of the BP-ANN model for prediction of organic coatings.

Artificial neural networks (ANNs) are adaptive and have parallel information-processing structures, which have the ability to build functional relationships between data and to provide powerful tools for non-linear, multidimensional interpolations. The back-propagation artificial neural network (BP-ANN) is a kind of artificial neural network model consisting of three adjacent layers: the input, hidden and output layers. Each layer may have several sub-layers and several processing elements. The structure of the BP-ANN used in this work is shown in Fig. 11. It is a three-layer network, which consists of an input layer with two input elements, an output layer with one output element and a hidden layer. A certain arrangement of the processing elements and links between layers produce a certain ANN model, which is suitable for certain tasks.

In the three-layer BP-ANN, if the input element of the input layer is *p*_j_, according to the neural network mapping relationship, the input and output elements of the hidden and output layers can be described in the mathematical formula (1)–(2):


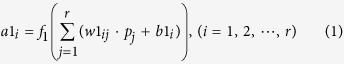



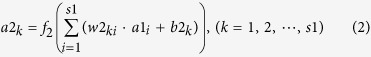


where *a*1_i_ and *a*2_k_ are the output elements of hidden layer and output layer, respectively; *f*_1_ and *f*_2_ are the transfer functions of hidden layer and output layer; *w*1_ij_ is the weight factor (probability of data transmission along a path) connecting input layer to hidden layer, and *w*2_ki_ is the weight factor connecting hidden layer to output layer; *b*1_i_ and *b*2_k_ are the bias values connecting input layer to hidden layer and hidden layer to output layer, respectively^34^,^35^.

BP-ANN learns from input-output samples to become clever i.e. capable of giving outputs based on inputs which it has not seen before. It consists of two parts: the feed-forward of information and the back-propagation of error. The learning process employs a learning algorithm, during which the BP-ANN develops a mapping function between the inputs and outputs. Basically, in a learning process, the input elements receive data from the external environment and pass them to the hidden elements. Some simple, yet, useful mathematical computations, which involve the weight factors of the connections and the input values, are applied during this process. The results from the hidden layer elements are mapped onto appropriate transfer function of each element and the final outputs are produced. And the error is then calculated by computing the difference between the network outputs and targets. Then the weight factors and the bias values of the adjacent layers will be modified according to the back-propagation of errors, which aims to get a smaller output error, and the computation processes are repeated throughout the layers. The entire training process is iterative, and stops when an acceptably small error is achieved. After completion of the learning process, the BP-ANN should be able to give output results for any given set of input data based on the generalized mapping that it has developed.

There are many algorithms available for the transfer function of the ANN model, and hyperbolic tangent sigmoid transfer function *tansig* 
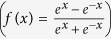
, log-sigmoid transfer function *logsig* 
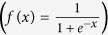
 and linear function *pureline (f*(*x*) = *x*) are the most frequently used among them^36^. The main task of the neural network is approximating the specified error by a non-linear function and making a prediction. Therefore, non-linear transfer functions should be selected between the layers to complete advanced processing. Here, log-sigmoid transfer function *logsig* is chosen as the transfer function both between the input layer and the hidden layer and between the hidden layer and output layer, that is, 
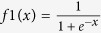
.

So the three-layer BP-ANN used in this work consists of a hidden layer of log-sigmoid transfer function followed by an output layer of log-sigmoid transfer function. The number of input elements to the proposed network is two and the number of elements in output layer is one. The size of hidden layer between input and output layers are determined according to the empirical formula *n* = 2 *m* + 1^22^, where *m* is the number of input elements and *n* is the element number in hidden layer. In our study, *m* is 2, and, so *n* is 5. Thus the network consists of two input elements, five elements at hidden layer and one output element, which is a 2 × 5 × 1 network.

### Sample data and validation data

One hundred sets of experimental data of the accelerated tests are applied and some of them are shown in Table 2. Ninety sets of data are divided into training samples and the other ten sets of data are validation samples.

In order to ensure that the data convergence is fast and accurate, the range of input (*p*) and output (*a*2) of BP-ANN network should fall within a certain range. So, the original data is normalized into the range of [0, 1], using formula (3):


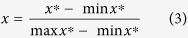


where *x* is the normalized data, x^*^ is the original data, min x^*^ and max x^*^ are the minimum and maximum of all original data, respectively.

### Training tests of the BP-ANN

The objective of the training process is to model the mapping between the input and the output without modeling the noise in the data. During the training process, based on the training data set including a set of input vectors and the corresponding output values, the weight factors and the bias values of the BP-ANN model are adjusted to minimize the errors between the model outputs and the targets. The performance function, or error function, is to be minimized during the training process. The typical performance function takes the following form:


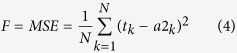


where *N* is the number of training input and output pairs, *t*_k_ and *a*2_k_ are the actual output (target) and the model output, respectively.

The training style is the supervised learning in which the rules are supplemented with a set of examples (the training set) of proper network behavior. The training set consists of inputs and the corresponding correct outputs (targets). There are several algorithms available for training BP-ANN, such as the standard back propagation algorithm and the Levenberg-Marquardt (LM) algorithm. If we choose the training functions with a slow speed of convergence, for example, *traingd* or *trainbfg*, it will take longer time to find suitable neural network. In our study, the training function *trainlm* utilizing the LM algorithm is taken, because of its fast convergence and advantage of avoiding falling into partial extremum^37^.

The validation data set is used to avoid the problem of “overfitting” during the ANN training. The “overfitting” phenomenon is that the error on the training set is driven to a very small value, but the error gets large when new data are presented to the trained network^19^. Data in the training set is used to adjust the weight factors and bias values, while the data in the validation set is used to verify the results. During the network training process, the error for the training set and that for the validation set will drop early in the training process, because the network is learning the relationship between the inputs and the outputs by modifying the trainable weights based on the training set. After a certain point, however, the error for the validation set will start to increase, because the network starts to model the noise in the training set. The training process can be stopped at this point, and a trained network with good modeling and generalization capability can be achieved.

Matlab R2011b Neural Network Toolbox was used to constitute the BP-ANN. In shortly, immersion time, pressure value and impedance modulus were named as P1, P2 and P3, respectively. When the service property prediction was expected, then the normalized results of P1 and P2 were utilized as input vectors of the neural network and the normalized results of P3 as output vector. When the service time prediction was expected, the normalized results of P2 and P3 were utilized as input vectors and the normalized results of P1 as output vector. The element number in hidden layer was five. Learning function *learngd* and performance function *MSE* were adopted. In training stage, epoch number and error goal were selected as 10,000 and 0.001, respectively.

## Additional Information

**How to cite this article**: Tian, W. *et al*. Lifetime prediction for organic coating under alternating hydrostatic pressure by artificial neural network. *Sci. Rep.* **7**, 40827; doi: 10.1038/srep40827 (2017).

**Publisher's note:** Springer Nature remains neutral with regard to jurisdictional claims in published maps and institutional affiliations.

## Figures and Tables

**Table 1:** Initial and final values of impedance parameters for coating samples immersed in 3.5 wt.% NaCl solution.

		|*Z*|(G Ω ·cm^2^)
Initial values (at 0 h)	*P* = 1 atm	40.7
	*P* = 35 atm	20.4
	*P* = 60 atm	72.4
	*P* = 81 atm	58.9
	*P* = 100 atm	38.9
Final values (at 240 h)	*P* = 1 atm	1.9
	*P* = 35 atm	0.49
	*P* = 60 atm	0.098
	*P* = 81 atm	3.68E-2
	*P* = 100 atm	1.23E-3

**Table 2:** The serving time, pressure values and impedance modulus of 100 sets of the accelerated tests.

Samples	Serving time (h)	Pressure values (atm)	log impedance modulus (log |*Z*|)
Training data			
1	3	1	10.460
2	36	1	9.604
3	108	1	9.561
4	204	1	9.455
5	2	35	10.020
6	72	35	8.960
7	168	35	8.855
8	232	35	8.375
9	12	60	10.234
10	108	60	8.382
11	198	60	8.186
12	5	81	10.400
13	48	81	9.052
14	156	81	7.464
15	12	100	9.822
16	120	100	5.674
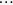	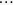	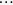	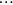
Validation data			
91	84	1	9.547
92	114	1	9.512
93	238	1	9.445
94	210	35	8.828
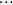	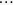	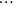	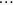

**Table 3:** The predicted data (predicted impedance modulus) and the original data (experimental impedance modulus) of the ten validation samples for serving property prediction of coatings.

Validation samples	Experimental data (log impedance modulus)	Predicted data (log impedance modulus)	Prediction accuracy
1	9.547	9.557	99.8%
2	9.512	9.547	99.6%
3	9.445	9.429	99.8%
4	8.828	8.670	98.2%
5	8.862	8.833	99.7%
6	7.512	7.476	99.5%
7	7.944	8.133	97.6%
8	7.529	7.461	99.1%
9	5.660	5.665	99.9%
10	5.953	5.955	99.9%

**Table 4:** The predicted data (predicted immersion time) and the original data (experimental immersion time) of the ten validation samples for life prediction of coatings.

Validation samples	Experimental data (immersion time)	Predicted data (immersion time)	Prediction accuracy
1	84	92	90.6%
2	114	143	75.0%
3	238	212	89.3%
4	210	151	72.0%
5	162	150	92.7%
6	198	168	84.9%
7	80	82	97.6%
8	138	164	80.6%
9	110	83	74.9%
10	188	171	90.8%
